# Clinical Efficacy of Traditional Chinese Medicine, Suan Zao Ren Tang, for Sleep Disturbance during Methadone Maintenance: A Randomized, Double-Blind, Placebo-Controlled Trial

**DOI:** 10.1155/2015/710895

**Published:** 2015-08-04

**Authors:** Yuan-Yu Chan, Yi-Hung Chen, Szu-Nian Yang, Wan-Yu Lo, Jaung-Geng Lin

**Affiliations:** ^1^Department of Psychiatry, Taoyuan Armed Forces General Hospital, No. 168, Zhong-Xing Road, Taoyuan 32551, Taiwan; ^2^Graduate Institute of Integrated Medicine, China Medical University, No. 91, Hsueh-Shih Road, Taichung 40402, Taiwan; ^3^Graduate Institute of Acupuncture Science, China Medical University, No. 91, Hsueh-Shih Road, Taichung 40402, Taiwan; ^4^Department of Biotechnology, Hung Kuang University, No. 1018, Sec. 6, Taiwan Boulevard, Shalu District, Taichung 43302, Taiwan; ^5^Graduate Institute of Chinese Medical Science, China Medical University, No. 91, Hsueh-Shih Road, Taichung 40402, Taiwan

## Abstract

Methadone maintenance therapy is an effective treatment for opiate dependence, but more than three-quarters of persons receiving the treatment report sleep quality disturbances. In this double-blind, randomized, controlled trial, we recruited 90 individuals receiving methadone for at least one month who reported sleep disturbances and had Pittsburgh Sleep Quality Index (PSQI) scores > 5. The purpose of this study was to determine whether Suan Zao Ren Tang, one of the most commonly prescribed traditional Chinese medications for treatment of insomnia, improves subjective sleep among methadone-maintained persons with disturbed sleep quality. Ninety patients were randomly assigned to intervention group (*n* = 45) and placebo group (*n* = 45), and all participants were analyzed. Compared with placebo treatment, Suan Zao Ren Tang treatment for four weeks produced a statistically significant improvement in the mean total PSQI scores (*P* = 0.007) and average sleep efficiency (*P* = 0.017). All adverse events (e.g., lethargy, diarrhea, and dizziness) were mild in severity. Suan Zao Ren Tang is effective for improving sleep quality and sleep efficiency among methadone-maintained patients with sleep complaints.

## 1. Introduction

Heroin dependence is one of the chronic medical illnesses and a public health problem worldwide. The social, medical, and economic consequences of heroin dependence are profound, including disrupted relationships, lost productivity, crime, violence, and the transmission of HIV/AIDS and other infectious diseases [1, 2]. Since the mid-1960s, long-term methadone maintenance therapy (MMT) has been the treatment of choice for heroin dependence [3–6] and has proven to be an effective long-term treatment for severe opiate dependence [7]. However, more than three-quarters of persons receiving MMT report sleep quality disturbances [8–10]. The severity of sleep symptoms has been associated with comorbid psychiatric disorders, chronic pain, lower levels of daily functioning, and the use of other drugs [9–11].

Methadone-maintained patients with sleep disturbances receive, on average, fewer than six hours of sleep daily [12]. A study from China found that 96.3% of methadone-maintained patients had Pittsburgh Sleep Quality Index (PSQI) scores ≥ 8 [13]. Other studies have reported that 84% of methadone-maintained patients have PSQI scores ≥ 6 [10], whereas another study found that only 35.2% of the general population had PSQI scores > 5 [14]. Generally, the incidence of sleep disturbances among methadone-maintained patients is significantly higher than that for the general population. Short sleep durations have been associated with daytime symptoms such as cognitive difficulties, reduced adherence to methadone treatment, and increased risk of relapse, physical injury, and motor-vehicle accidents [15, 16]. Two polysomnographic studies show reduction of rapid eye movement (REM) sleep and slow wave sleep during induction and maintenance of opioid use [17, 18].

Sleep-disordered breathing, including obstructive sleep apnea (OSA) and central sleep apnea (CSA), is another potential factor leading to disrupted sleep in MMT patients. Some studies showed that CSA has been reported to occur in 30–60% of MMT patients [19, 20] and associated with methadone dose and concomitant benzodiazepine use [21].

In one study of 71 OSA and CSA patients who were in MMT at least 3 months, the results showed that CSA was not associated with methadone doses and OSA was more common than CSA in this population. OSA was associated with higher body mass index, longer duration in MMT, and non-Caucasian race. Moreover, subjective sleep disturbance measured with the PSQI was not related to OSA or CSA [12].

There are some mechanisms that may explain insomnia among methadone-maintained patients. First, opioids decrease REM sleep by decreasing acetylcholine release in the pontine reticular formation [22]. Second, opioids promote wakefulness by suppressing inhibitory gamma-aminobutyric acid (GABA) transmission in the dorsal raphe nucleus [23]. Third, sleep disturbances in MMT patients are associated with opioid-induced reduction of the nucleoside adenosine in the basal forebrain [24, 25].

In Western medicine, therapy for insomnia is mainly based on prescribed medications such as benzodiazepines (BZD), anticonvulsants, antidepressants, or over-the-counter antihistamines [26]. These medications, especially BZD, are sometimes associated with adverse effects and are not suitable for long-term use due to an increased risk of life-threatening multiple drug overdoses. In opiate-dependent patients, the misuse and abuse of BZD is a public health problem because methadone and BZD both have sedating effects on the central nervous system, which lead to difficulty in breathing as well as cognitive impairment [27, 28]. A recent survey revealed a 47% prevalence of lifetime use of BZD among methadone-maintained patients [29] and nonprescribed BZD use among MMT patients ranged from 44 to 66% [30, 31]. In addition, methadone-maintained patients who abuse BZD are associated with higher rates of depression and anxiety [32], increased risk of continuing opiate abuse, and discontinuing methadone treatment [33, 34].

Traditional Chinese medicine (TCM) has been widely used for centuries in Asian countries and is one of the most common complementary and alternative therapies used in Taiwan [35–38]. Previous studies have shown that TCM treatment is effective in improving sleep quality and prolonging sleep duration, and it is associated with fewer side effects (e.g., lethargy, dry mouth, and dizziness) than Western medicines [39–41].

In Taiwan, the National Health Insurance program has found that Suan Zao Ren Tang (SZRT) was the most commonly prescribed Chinese herbal formula for the treatment of insomnia [42]. SZRT was also the most common ingredient found in over-the-counter sleeping pills [43]. SZRT has been used to treat insomnia for centuries, as noted in the ancient Chinese book* Synopsis of Prescriptions of the Golden Chamber*, by Chang Chung Ching (AD 150–219). In classical literature, SZRT is said to bring on a tranquillizing sensation and assists in alleviating insomnia, restlessness, palpitations, and mental distress. In 2007, Yi et al. found SZRT was shown to increase non-REM sleep in an experimental model in rats, and the mechanism was thought to involve the stimulation of GABA_A_ and serotonin receptors [44, 45].

Systematic reviews of complementary and herbal medicine for insomnia have been published recently [46–48], but these reviews found that a majority of the randomized clinical trials on TCM were of poor methodological quality. However, SZRT has never been tested in opiate-dependent patients. Therefore, we have designed a randomized, double-blind, placebo-controlled clinical trial to test whether SZRT is effective in improving sleep quality, sleep latency, sleep duration, sleep efficiency, anxiety, and depression among methadone-maintained patients with sleep complaints.

## 2. Methods

### 2.1. Participants

Patients were recruited from the outpatients of the Department of Psychiatry at Taoyuan Armed Forces General Hospital, a regional teaching hospital in Taiwan, between August 2013 and December 2013. Ninety patients were recruited for the study and divided into an intervention group and a placebo group.

Patients eligible for inclusion in the study were over 20 years of age; had fulfilled the criteria for opiate dependence (Diagnostic and Statistical Manual of Mental Disorders, Fourth Edition); had been receiving MMT for more than one month; had reported a sleep disturbance with a PSQI score > 5; and provided signed informed consent. Individuals were excluded if they had received any antidepressant or neuroleptic medication; had received any TCM treatment during the previous 30 days; had any serious physical or mental illness; exhibited a significant risk of suicide; were pregnant; or were unable to read and fill out the forms for the study.

### 2.2. Study Design and Procedure

The study was conducted as a randomized, double-blind, placebo controlled trial to examine the effects of SZRT in methadone-maintained persons with sleep complaints. All participants were randomly assigned to an intervention or placebo group in a ratio of 1 : 1 using computer generated random numbers without stratification by background characteristics. The random number list was prepared by an investigator with no clinical involvement in the trial. The allocation sequence was concealed from the researcher enrolling and assessing participants in sequentially numbered, opaque, sealed, and stapled envelopes. Corresponding envelopes were opened only after the enrolled participants completed all assessments and intervention. The research staff and participants were blinded to their assignment. The study procedures and informed consent form were approved by the Institutional Review Board of Tri-Service General Hospital National Defense Medical Center in Taiwan (IRB number TY101-18) and registered in http://www.clinicaltrials.gov/ (NCT01913418).

### 2.3. Herbal Formula

In a typical Chinese herbal medicine prescription, a complex integration of two or more single Chinese herbs is formulated together to achieve additive or synergistic effects. SZRT is composed of the following five herb ingredients: Semen Zizyphi Spinosae (Suanzaoren), Sclerotium Poriae Cocos (Fuling), Radix Ligustici Chuanxiong (Chuanxiong), Rhizoma Anemarrhena (Zhimu), and Radix Glycyrrhizae (Gancao). The SZRT formula used in this study was manufactured as a herbal extract powder using the good manufacturing procedures of the certified company Kaiser Pharmaceutical Co., Ltd. (Taiwan). The granules were packed in aluminum foil packages and administered orally at a dose of 4 g, three times per day for four weeks. Kaiser Pharmaceutical was not involved in any other sponsorship, study design, or monitoring of the participants. The placebo granules were prepared with 4 g starch inside foil packages of the same color and size as the SRZT. Thus, neither the investigators nor patients knew the group to which a patient was allocated.

### 2.4. Intervention

The participants were provided either SZRT or placebo and the intervention period was four weeks. We selected the intervention period because of a previous study that reported a significant improvement of sleep quality after four weeks of SZRT treatment [49]. In each week, patients were provided with a one-week supply of SZRT or placebo. No other medication or behavioral therapy to address the sleep disturbance was allowed during the study period. After four weeks on the prescribed medication, the participants discontinued the use of SZRT or placebo.

### 2.5. Measures

Patient questionnaires included basic data such as gender, age, marital status, occupation, recent heroin use, recent amphetamine use, history of heroin use, and history of amphetamine use. The patients completed the questionnaire at baseline (first visit) and after four weeks (second visit). In this study, all assessments were completed by the same rater who was blind to the treatment allocation.

The primary outcome measure under investigation was sleep quality. Sleep quality was assessed using the Taiwanese version of the PSQI [50], which has demonstrated reliability and validity [51]. This system evaluates sleep disturbances in seven subscales: subjective sleep quality, sleep latency, sleep duration, habitual sleep efficiency, sleep disturbances, use of sleeping medication, and daytime dysfunction. Each subscale is rated on a 4-point scoring system (0 to 3, with 3 indicating a more profound effect), and scores from the subscales are summed together to yield a global score (0 to 21). Higher scores indicate a greater severity of sleep disturbance, and a global score > 5 is indicative of “poor sleep” [50].

Secondary outcome measures include one-week sleep diaries, severity of Beck anxiety inventory (BAI) score, Beck Depression Inventory II (BDI-II, second edition) score, and heroin craving. Participants completed a daily morning sleep diary during the week in which they recorded bedtime, time to fall asleep, number of awakenings, time awake during the night, and wake-up time. Total time in bed was calculated as the duration between bedtime and wake-up time. Diary total sleep time (TST) was calculated by subtracting sleep onset latency (SOL) and time awake during the night from diary time in bed. Diary sleep efficiency (SE) was calculated by dividing diary total sleep time by total time in bed × 100. Each sleep measure was averaged over the reported days. We included participants with 3 to 7 diary days because sleep diary analyses in other populations indicate that reliable sleep estimates can be obtained with ≥3 days of data [52].

The severity of anxiety symptoms was measured using the BAI [53]. This measure lists 21 symptoms of anxiety, such as feeling hot, scared, or nervous. Participants were instructed to rate the extent to which they were bothered by each of these symptoms over the previous week. Each item could be rated on a 4-point Likert scale, ranging from 0 (not bothered) to 3 (severely bothered), yielding a maximum total score of 63 points. The BAI manual considers total scores of 0 through 7 minimal anxiety, 8 through 15 mild anxiety, 16 through 25 moderate anxiety, and 26 through 63 severe anxiety.

Depression symptoms were measured using the BDI-II. This measure consists of 13 items, rated from 0 to 3, to evaluate depression according to the degree to which it reflected the patient's state during the previous week. The BDI-II has a high reliability and concurrent validity and the BDI-II manual considers total scores of 0 through 13 minimal depression, 14 through 19 mild depression, 20 through 28 moderate depression, and 29 through 63 severe depression [54].

The severity of heroin craving was assessed using a 100-millimeter visual analog scale (VAS). The amount of heroin craving experienced by a participant can range across a continuum from no craving to extreme craving. The VAS score was determined by measuring in millimeters from the left-hand end of the line to the point that the patient marks [55].

### 2.6. Statistical Analysis

Statistical analyses were based on intention-to-treat model. Missing data was handled using last observation carried forward imputation technique. SPSS (version 18.0) was used for statistical analysis. Baseline differences and the treatment effects between the SZRT and the placebo group were examined using either unpaired *t*-tests or *χ* ^2^ tests.

Our sample size estimation was based on changes in PSQI score, the primary outcome measure. Based on a previous SZRT study [49], a clinically significant treatment effect was defined as ≥2 point difference in PSQI score between treatment groups, and the effect size was 0.667. G*∗*Power 3.1.3 was used for *t*-tests to compute that a sample of 37 individuals per group would have a power of 80% to differentiate between groups at an alpha level 0.05. Allowing for a 20% attrition rate, we estimated this study would require a sample size of 45 patients per group.

## 3. Results

### 3.1. Patient Characteristics

**Figure 1:**
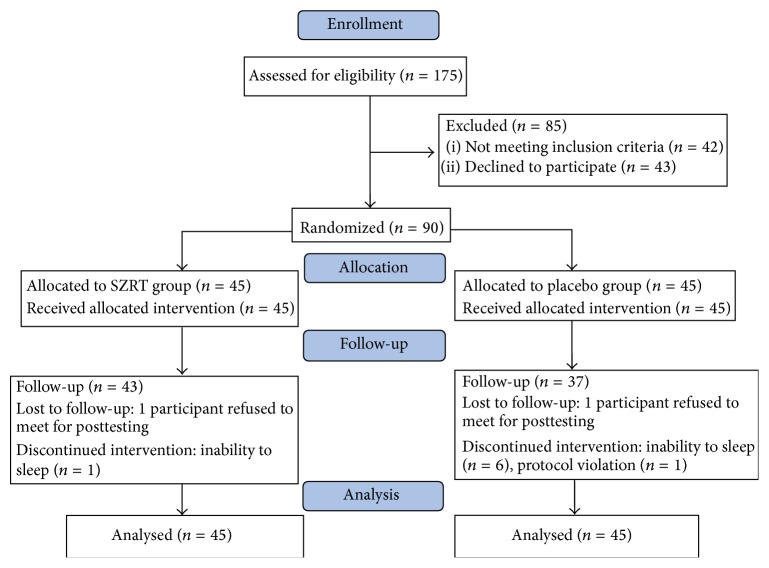
The diagram specifics the flow of participants through the enrollment, allocation, follow-up, and analysis phases of the RCT study.

Of 175 potential patients assessed for eligibility, 85 declined participation or failed to meet inclusion criteria (Figure 1). Ninety patients were randomly assigned to the intervention group (*n* = 45) and the placebo group (*n* = 45), while 80 (88.9%) of them completed the 4-week treatment. The mean age of participants was 39.6 years, and 80% were male (Table 1). The mean PSQI total score was 11.5 ± 3.1. Mean methadone dose at baseline was 55.7 ± 31.6 mg. Heroin and amphetamine abuse histories averaged 9.4 ± 5.7 and 5.5 ± 6.5 years, respectively. In the 30 days prior to baseline, 53.3% and 20% of participants reported using heroin or amphetamine, respectively. Intervention groups did not differ significantly (*P* > 0.05) in any of the baseline characteristics described in Table 1. Seven subjects (7.8%) discontinued intervention within the three weeks due to a complaint of no response after treatment. One subject (1.1%) violated the protocol due to severe alcohol drinking and using other medication. Two subjects (2.2%) lost to follow-up because they refused to meet for posttesting (Figure 1).

### 3.2. Efficacy

Table 2 shows baseline means, follow-up means, and changes in mean subjective sleep parameters by intervention group. The result shows that between baseline and week 4, mean total PSQI scores decreased by 3.8 and 1.9 points, and average SE increased by 11.4% and 5.0% among those randomized to SZRT and placebo, respectively. Compared with placebo treatment, Suan Zao Ren Tang treatment for four weeks produced a statistically significant improvement in the mean total PSQI scores (*P* = 0.007) and average SE (*P* = 0.017). In the SZRT group, the average time to fall asleep per day decreased from 41.5 minutes at baseline to 26.5 minutes after treatment. The average total sleep time per day increased from 342.6 minutes at baseline to 412.8 minutes after treatment.

Table 3 presents the mean values for secondary outcomes by treatment condition at baseline and after treatment. After treatment, BAI and BDI scores in both groups improved from the baseline. However, BAI, BDI, and heroin craving scores did not differ significantly between the groups after treatment.

### 3.3. Adverse Events

In the SZRT group, diarrhea (*n* = 2), sweating (*n* = 2), dizziness (*n* = 1), and morning sleepiness (*n* = 1) were reported as adverse events. In the placebo group, adverse events included morning sleepiness (*n* = 2) and headache (*n* = 1). Most adverse events were mild in severity.

## 4. Discussion

To the best of our knowledge, this is the first randomized, placebo-controlled trial testing SZRT for opiate-dependent persons, a population with extraordinarily high rates of sleep disturbances. Some studies suggest that poor-quality sleep is a universal risk factor for drug relapse [56], and it is possible that there are overlapping neurobiological mechanisms common to sleep disorders, depression, and anxiety [57, 58]. Our results demonstrate that SZRT improves subjective sleep quality and sleep efficiency in methadone-maintained persons with sleep disturbances. But the PSQI score was still more than five at the end of treatment; long-term treatment effect should be investigated in the future.

In our study, we found that the chance of dropping out was higher for patients treated with placebo than for those treated with SZRT. There are six patients discontinued intervention because of inability to sleep in the placebo group. However, only one patient discontinued intervention because of the same reason in the SZRT group. A previous meta-analysis reported that attrition rate may be higher in the placebo arm than in the active treatment arm of a placebo-controlled trial because of the lack of efficacy of placebo, causing patients to leave the study prematurely [59].

Regarding basic research into the treatment of insomnia, current pharmacological approaches focus primarily on GABA, the major inhibitory neurotransmitter in the central nervous system [60]. SZRT was shown to increase non-REM sleep in an experimental model in rats, and the mechanism was thought to involve the stimulation of GABA_A_ and serotonin receptors [44, 45]. Suanzaoren (*Z. spinosa*) was the chief ingredient of SZRT. It has been reported to have a sedative effect at higher doses and an anxiolytic effect at lower doses in animal studies [61]. In addition, there are some components of Suanzaoren that may have sedative-hypnotic effects. Jujuboside A is a main component of jujubogenin extracted from the seed of Suanzaoren, which produces its sedative-hypnotic effects probably through its anticalmodulin action and inhibiting the glutamate-mediated excitatory signaling pathway in the hippocampus [62]. Saponins, isolated from Suanzaoren, can prolong the sleep time induced by barbiturates [63]. Sanjoinine A is one of major alkaloid compounds of Suanzaoren, which have anxiolytic-like effects, increase sleep time, and decrease sleep latency induced by pentobarbital, and these effects may be mediated by GABAergic transmission [64, 65]. Other ingredients of SZRT, including Fuling (*P. cocos*), have been reported to enhance non-REM sleep by enhancing the secretion of cytokines, such as interleukin-1*β* and tumor necrotic factor-*α*, in human peripheral blood monocytes [66, 67]. Gancao* (liquorice extract)* has been shown to have an antidepressant-like effect that seems to be mediated by increase of brain norepinephrine and dopamine, but not by increase of serotonin in a murine model [68]. In a clinical trial, SZRT appeared to be a relatively safe and effective short-term therapeutic option for improving daytime function of climacteric women with poor sleep quality [49].

In 2012, two studies [69, 70] reported the treatment for methadone-maintained patients with sleep disturbance. The baseline of the mean PSQI total score in the two studies [69, 70] was similar to our study. Li et al. [69] used acupuncture therapy for their patients. Acupuncture was applied to Baihui (GV20), Shenmen (bilateral, TF4), Shenting (GV24), Sanyinjiao (bilateral, SP6), and Sishencong (EX-HN1). They found that acupuncture treatment produced a statistically significant improvement in the mean total PSQI scores after four weeks of treatment [69]. Stein et al. reported trazodone did not improve subjective or objective sleep in methadone-maintained patients with sleep disturbance [70]. In addition, a previous study evaluated the effectiveness of melatonin in attenuating sleep difficulties during BZD withdrawal among patients in methadone maintenance treatment [71]. They found that melatonin improved sleep quality in patients who did not stop BZD but did not enhance BZD discontinuation [71].

Psychiatric comorbidity between anxiety, depression, and substance abuse is common. The prevalence of psychiatric disorder is up to 10 times higher in the methadone-maintained patients than in the general population [72]. At least one anxiety disorder in 55% and affective disorder in 58% were found in the population receiving methadone maintenance treatment [73]. In our study, the average BAI scores in the baseline and posttreatment were 15.0 and 12.8 which both indicated mild anxiety. The average BDI-II scores in the baseline and posttreatment were 18.3 and 13.0 which indicated mild depression in the baseline and minimal depression after treatment. In Asia, Suanzaoren has been prescribed for the treatment of depression, insomnia, and anxiety. The possible mechanism of action involves the serotonergic, noradrenergic, and monoamine oxidase enzyme system [61, 74]. However, both BAI and BDI-II scores did not differ significantly between the groups after treatment in our study. Long-term treatment and bigger sample size for assessing the antidepressant and anxiolytic effect of SZRT should be further investigated.

A major limitation of our study is lack of an objective measurement, such as polysomnography. A previous study comparing subjective ratings, sleep diaries, and home polysomnography in methadone-maintained patients showed that subjective and objective sleep durations were similar in their sample, and average diary sleep time, subjective ratings of feeling rested, and polysomnography sleep efficiency were correlated significantly with PSQI score [75]. Therefore, the subjective measures used in this study may be indicative of objective sleep measures in methadone-maintained patients. The study is also limited by participant self-reporting, which may exaggerate the severity of sleep problems. Another limitation is the sleep efficiency calculation; typically sleep efficiency comparisons are based on a standard time in bed values, generally 480 minutes in sleep laboratory studies. A longer or shorter total time in bed can result in different sleep efficiency. In our study, we do not receive a standardized time in bed. At baseline of the total time in bed, the SZRT group was less than placebo group more than 30 minutes. In the 4-week posttreatment assessment, the SZRT group still had shorter total time in bed than the placebo group. But there was no significant difference between two groups in the total time in bed. However, a shorter total time in bed can result in a relatively high sleep efficiency. This difference complicates the sleep efficiency comparison and the claim of a significant difference. In addition, participants were all from Taiwan; there needs to be caution in generalizing these findings to patients in other nations. Finally, although patients provided self-reports regarding current illicit drug use, the validity of these reports was not confirmed with urine toxicological data. Methadone-maintained patients have many ongoing risks for chronic insomnia and have high rates of use of other illicit drugs (e.g., amphetamine), cigarettes, or alcohol that can interfere with sleep.

Despite the limitations, this study retains a number of noteworthy strengths. The use of TCM for insomnia can be traced back more than 2000 years in Chinese medical texts. Notwithstanding the popularity of SZRT in the markets of China, Japan, South Korea, and Taiwan, this is the first evaluation of SZRT in opiate-dependent patients by a double-blind, placebo-controlled, randomized clinical trial. The 4-week courses of the SZRT treatment show it to be a well-tolerated and valuable alternative therapeutic option for improving sleep quality in methadone-maintained patients.

## 5. Conclusion

Our data demonstrate that SZRT is effective in improving sleep quality and sleep efficiency among methadone-maintained patients with sleep complaints and appears safe in combination with methadone.

## Figures and Tables

**Table 1:** Demographic and clinical characteristics of the participants.

Variable	SZRT (*n* = 45)	Placebo (*n* = 45)	Total (*n* = 90)	*χ* ^2^/*t* value^a^	*P* value
Age (years)	40.6 ± 7.2	38.6 ± 6.9	39.6 ± 7.1	1.34	0.18
Gender (male)	34 (75.6%)	38 (84.4%)	72 (80%)	1.11	0.29
Duration of methadone maintenance (months)	25.4 ± 19.8	19.6 ± 19.7	22.5 ± 19.8	1.40	0.17
Recent heroin use (yes)	24 (53.3%)	24 (53.3%)	48 (53.3%)	0.00	1.00
Recent amphetamine use (yes)	11 (24.4%)	7 (15.6%)	18 (20%)	1.11	0.29
Heroin use (years)	8.8 ± 5.7	9.9 ± 5.8	9.4 ± 5.7	−0.84	0.40
Amphetamine use (years)	4.9 ± 6.0	6.1 ± 6.9	5.5 ± 6.5	−0.85	0.40
Methadone dosage (mg)	58.9 ± 32.5	52.4 ± 30.6	55.7 ± 31.6	0.97	0.34
PSQI total scores	11.6 ± 3.6	11.5 ± 2.5	11.5 ± 3.1	0.10	0.92
Total time in bed (min)	446.6 ± 104.2	489.8 ± 111.5	468.2 ± 109.5	−1.90	0.06
Total sleep time (min)	342.6 ± 95.2	377.0 ± 101.1	359.8 ± 99.2	−1.66	0.10
Sleep efficiency (%)	76.5 ± 11.3	79.0 ± 11.1	77.8 ± 11.2	−1.05	0.30
Sleep onset latency (min)	41.5 ± 36.7	38.8 ± 18.9	40.1 ± 29.0	0.45	0.66
BAI	15.0 ± 11.3	12.4 ± 13.9	13.7 ± 12.6	0.96	0.34
BDI-II	18.3 ± 14.3	14.4 ± 14.2	16.4 ± 14.4	1.27	0.21
Heroin craving score (mm)	25.3 ± 28.1	33.7 ± 34.6	29.6 ± 31.6	−1.22	0.22

Note: data are presented as mean ± SD or number (%). ^a^Comparison between SZRT group and placebo group by *χ* ^2^ or unpaired *t*-test.

PSQI, Pittsburgh Sleep Quality Index; BAI, Beck Anxiety Inventory; BDI-II, Beck Depression Inventory II.

**Table 2:** Mean and change in mean subjective sleep parameters.

	SZRT group (*n* = 45)		Placebo group (*n* = 45)		*P* value^a^
	Mean (SD)	Change	Mean (SD)	Change	
PSQI					
Total scores					
Baseline	11.6 (3.6)		11.5 (2.5)		
4-week posttreatment	7.8 (3.7)	−3.8	9.6 (3.1)	−1.9	0.007^*∗*^
Sleep diary					
Total time in bed					
Baseline	446.6 (104.2)		489.8 (111.5)		
4-week posttreatment	466.3 (78.8)	24.1	481.7 (113.6)	−5.5	0.261
Mean TST					
Baseline	342.6 (95.2)		377.0 (101.1)		
4-week posttreatment	412.8 (81.3)	70.2	418.8 (121.9)	41.8	0.228
Mean SL					
Baseline	41.5 (36.7)		38.8 (18.9)		
4-week posttreatment	26.5 (18.3)	−15.0	31.8 (31.5)	−6.9	0.211
Mean SE (%)					
Baseline	76.5 (11.3)		79.0 (11.1)		
4-week posttreatment	87.9 (9.0)	11.4	84.1 (15.4)	5.0	0.017^*∗*^

^a^Difference between mean change scores SZRT and placebo groups (*t*-test). TST, total sleep time; SL, sleep latency; SE, sleep efficiency.

^*∗*^ *P* < 0.05.

**Table 3:** Mean and change in BAI, BDI, and heroin craving score.

	SZRT group (*n* = 45)		Placebo group (*n* = 45)		*P* value^a^
	Mean (SD)	Change	Mean (SD)	Change	
BAI					
Baseline	15.0 (11.3)		12.4 (13.9)		
4-week posttreatment	12.8 (13.2)	−2.2	8.8 (13.0)	−3.6	0.502
BDI					
Baseline	18.3 (14.3)		14.4 (14.4)		
4-week posttreatment	13.0 (13.9)	−5.3	11.5 (13.3)	−2.9	0.280
Heroin craving score					
Baseline	25.5 (28.1)		33.7 (34.6)		
4-week posttreatment	33.5 (31.9)	8.3	34.2 (35.6)	0.68	0.351

^a^Difference between mean change scores SZRT and placebo groups (*t*-test).

## Abbreviations

MMT: Methadone maintenance therapy
PSQI: Pittsburgh Sleep Quality Index
REM: Rapid eye movement
BZD: Benzodiazepines
TCM: Traditional Chinese medicine
SZRT: Suan Zao Ren Tang
GABA: Gamma-aminobutyric acid
BAI: Beck Anxiety Inventory
BDI-II: Beck Depression Inventory II
TST: Total sleep time
SOL: Sleep onset latency
SE: Sleep efficiency
VAS: Visual analog scale.
